# Extracorporeal Membrane Oxygenation as Life Support in Neonatal Respiratory Failure: A Single-Center Cohort Study and a Systematic Review

**DOI:** 10.3390/children11121441

**Published:** 2024-11-26

**Authors:** Raffaele Falsaperla, Rosanna Zanai, Ausilia Desiree Collotta, Vincenzo Sortino, Giovanna Vitaliti, Carla Cimino, Bruna Scalia, Marco Simone Vaccalluzzo, Michela Spatuzza, Grete Francesca Privitera, Alfredo Pulvirenti, Piero Pavone, Martino Ruggieri, Andrea Marino, Salvatore Agati

**Affiliations:** 1 Neonatal Intensive Care Unit, Azienda Ospedaliero Universitaria Policlinico “G. Rodolico-San Marco”, San Marco Hospital, University of Catania, 95121 Catania, Italy; carla.cimino87@gmail.com (C.C.); b.scalia@hotmail.it (B.S.); 2 Cardiovascular Department, Mediterranean Pediatric Cardiology Center, Bambino Gesù Children’s Hospital, 98035 Taormina, Italy; rosanna.zanai@virgilio.it (R.Z.);; 3 Pediatrics and Pediatric Emergency Operative Unit, Azienda Ospedaliero Universitaria Policlinico “G. Rodolico-San Marco”, San Marco Hospital, University of Catania, 95121 Catania, Italy; ausilia.collotta.92@gmail.com (A.D.C.); sortino.vinci@gmail.com (V.S.); giovitaliti@yahoo.it (G.V.); 4 Postgraduate Training Program in Pediatrics, Department of Clinical and Experimental Medicine, University of Catania, 95123 Catania, Italy; 5 Department of General Surgery and Medical Surgical Specialties, Section of Orthopaedics, A.O.U. Policlinico Rodolico-San Marco, University of Catania, 95123 Catania, Italy; marcovaccalluzzo@hotmail.it; 6 Institute for Biomedical Research and Innovation (IRIB), National Research Council, 98164 Catania, Italy; michela.spatuzza@gmail.com; 7 Bioinformatics Unit, Department of Clinical and Experimental Medicine, University of Catania, 95123 Catania, Italy; greteprivitera@gmail.com (G.F.P.); alfredo.pulvirenti@unict.it (A.P.); 8 Unit of Clinical Pediatrics, Department of Clinical and Experimental Medicine, AOU Policlinico “G. Rodolico-San Marco”, University of Catania, 95123 Catania, Italy; martino.ruggieri@unict.it; 9 Unit of Infectious Disease, Department of Clinical and Experimental Medicine, AOU Garibaldi, University of Catania, 95123 Catania, Italy; andrea.marino@unict.it

**Keywords:** extracorporeal membrane oxygenation∙neonatal, ECMO respiratory failure, life-support

## Abstract

Background: Extracorporeal membrane oxygenation (ECMO) is a life support in newborns with severe respiratory failure. Our main objective was to evaluate the mortality of patients and define positive and negative predictive factors of survival. Methods: We performed a Strengthening the Reporting of Observational Studies in Epidemiology (STROBE)-conformed retrospective observational study and a systematic review, according to the Preferred Reporting Items for Systematic Reviews and Meta-Analyses (PRISMA). Our data were analyzed using R (v.4.2.1). We performed survival analysis, correlation analysis, and Fisher’s exact test. The first endpoint was the mortality rate. The second endpoint was to evaluate all factors associated with survival. The third endpoint was focused on complications of ECMO. Results: Our study included 8 patients treated in our centers and 45 patients from the literature review. Survival was 79%. Positive predictive factors of survival were a length of ECMO of less than 10 days and male neonates, while prematurity and the presence of 2 complications were negative predictive factors. Conclusions: ECMO functions as life support, although mortality and morbidity risks are high.

## 1. Introduction

Extracorporeal life support (ECLS), also known as extracorporeal membrane oxygenation (ECMO), is a specialized form of cardiopulmonary bypass utilized as a rescue therapeutic intervention, notably beneficial for neonates with severe respiratory failure [1,2,3,4,5,6,7]. Its inaugural application for this demographic dates back to the 1970s, with Bartlett et al. pioneering its use for a neonate with a pronounced case of meconium aspiration syndrome (MAS), in whom ECMO was initiated for persistent fetal circulation [1].

Subsequent to this foundational implementation, myriad prospective and randomized investigations have corroborated the efficacy of ECMO in enhancing survival rates in neonates with acute respiratory distress, especially when juxtaposed with conventional medical treatment protocols. However, it is imperative to clarify that ECMO has not been universally embraced as a standard practice in neonatal intensive care. The contention in the manuscript about its widespread acceptance is not accurate [5]. Additionally, while the Extracorporeal Life Support Organization (ELSO) international registry serves as an invaluable repository of data pertaining to ECMO patients, it does not act as a primary source of knowledge on ECMO application. Specifically, the ELSO registry’s primary function is to amass data on patients undergoing ECMO [8]. The report from the ELSO registry published in August 2022 recorded data on 47,295 neonates, of which 34,432 were administered ECMO for respiratory failure. This registry documented a 72% survival rate among neonates who were subjected to ECMO due to respiratory complications. Evidently, neonatal respiratory ECMO has substantially ameliorated survival rates and fostered standard neurodevelopmental outcomes across various pathological conditions, inclusive of MAS and persistent pulmonary hypertension (PPHN) [9,10,11].

In our retrospective single-center cohort study, we juxtaposed our cohort of neonates supported by respiratory ECMO against the existing literature, conducting a systematic examination. Our main objective was to evaluate mortality in newborns undergoing respiratory ECMO and define any positive and negative predictors of survival. We also focused our analysis on complications as a further endpoint of our study. 

## 2. Materials and Methods

Our study was a retrospective single-center cohort study from April 2019 to June 2022. This study conformed to the Strengthening the Reporting of Observational Studies in Epidemiology (STROBE) statement, and all points on the checklist were respected. The included newborns were admitted to the Neonatal Intensive Care Unit of the San Marco University Hospital (Catania, Italy) and San Vincenzo Hospital of Taormina in the last 3 years and underwent ECMO. The clinical data were collected by accessing the patients’ hospital records.. Each parent expressed their consent for participation in the study and the publication of their data in anonymous form. The study was approved by the Ethical Committee of the University of Catania. 

## 3. Systematic Review

A comprehensive and systematic review was undertaken by querying both the PubMed MEDLINE and Embase databases in accordance with the guidelines delineated in the Preferred Reporting Items for Systematic Reviews and Meta-Analyses (PRISMA) statement and following the STROBE Checklist for accuracy (as shown in the Supplementary File). The search strategy deployed keywords and phrases such as “ECMO”, “extracorporeal membrane oxygenation”, “ECLS”, and “Extracorporeal Life Support” in conjunction with the term “respiratory” [12]. To refine the search output, specific filters were applied, limiting the results to articles written in English (reason 1) and focused on the newborn population. All identified articles were restricted to those published on or before 12 November 2022.

In the initial screening process, various types of articles were identified, including case reports, case series, letters to the editor, and review papers. From this collection, articles were excluded based on the following criteria: the presence of duplicates (reason 2), unavailability of the complete text (reason 3), narrative pieces, systematic reviews, and editorials (reason 4). Moreover, studies that sourced their data from the ELSO register were discarded due to potential case duplications and the absence of detailed anamnestic, clinical, and instrumental data, which would render our analysis both redundant and inconclusive (reason 5).

**Figure 1:**
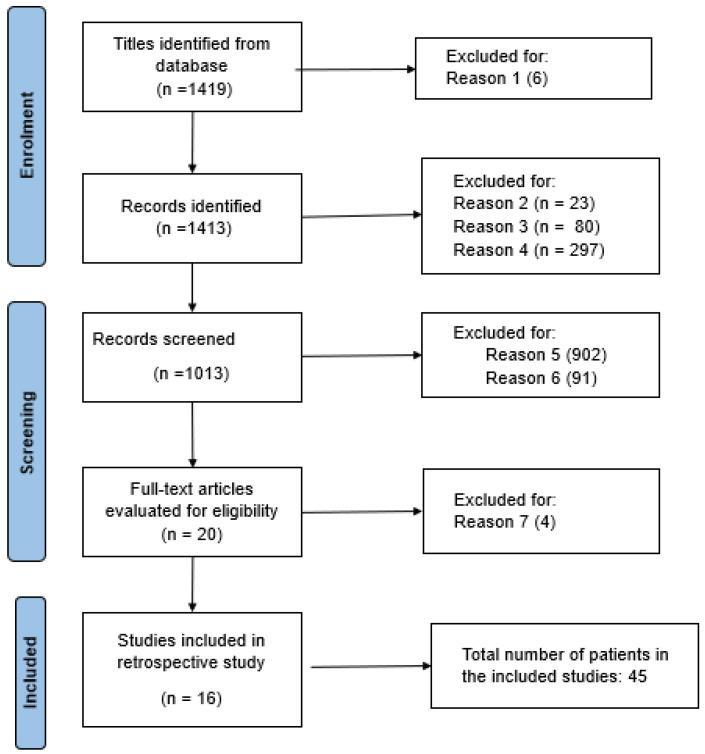
Research methodology of the systematic review according to the guidelines delineated in the Preferred Reporting Items for Systematic Reviews and Meta-Analyses (PRISMA) statement, with inclusion and exclusion criteria.

We included studies that provided details on parameters such as the age of the patients at the commencement of treatment, the underlying cause of respiratory failure, the duration of ECMO usage, the specific type of ECMO employed, survival rates, and any complications that arose post-ECMO therapy. A detailed flow diagram encapsulating the entire search and screening methodology is illustrated in Figure 1.

Patient eligibility was based on the following criteria: (1) less than 28 days of age or corrected age less than or equal to 40 weeks of gestation; (2) severe but potentially reversible respiratory failure unresponsive to standard therapy.

The absolute contraindications are the same adapted from the ELSO Guidelines for Neonatal Respiratory Failure 2017 [13] and ELSO Guidelines for Pediatric Cardiac Failure 2021 [14].

To ensure a rigorous and relevant assessment, works that did not align with our study’s primary objectives were excluded (reason 6). Additionally, studies devoid of specific clinical details pertaining to individual patients were not considered (reason 7). To augment our primary search results, references within the identified articles were meticulously examined to unearth any additional pertinent publications. All the acquired full texts were scrutinized by the same set of authors. The team collaboratively extracted data, which were then debated and evaluated to ensure the consistency of quality indicators and overall reliability.

## 4. Data Analysis

Clinical information retrieved from the database of selected patients and from the literature included anamnestic, clinical, and instrumental data and vital signs. Each patient was assigned a number from 1 to 8 to ensure anonymity. All the authors accurately reviewed the medical records and full texts of the works included in the research and the data were extrapolated and discussed within the group.

## 5. Bioinformatic Analysis

Our data were analyzed using R (v.4.2.1). We performed survival analysis, correlation analysis, and Fisher’s exact test. Survival probability was calculated using the time of ECMO as the endpoint of survival. The R packages survminer [15] and survival [16] were employed to calculate the survival probability and the associated *p*-value and to plot the survival curves. Fisher’s exact test was used to compare variables including the etiology of respiratory failure and prematurity with other types of comorbidity. The results were considered significant with a *p*-value < 0.05. The test results were plotted using ggstatsplot [17]. Furthermore, correlation analysis was conducted using Pearson’s correlation. Pearson’s correlation coefficient (R) can reach a value between −1 (total negative correlation) and 1 (total positive correlation). Each correlation was plotted via the package ggpubr (v.0.6.0) with a scatter plot to show the linear relationship between variables [18].

3.3. Endpoint

The first endpoint was the overall mortality rate in newborns who underwent ECMO treatment for respiratory failure. The second endpoint was the survival rate in relation to the duration of treatment, anamnestic data (gender, gestational age, etc.), the cause of respiratory failure, and the presence of comorbidities. The third endpoint focused on complications of ECMO, incidence, and contributory factors to mortality.

## 6. Results

### 6.1. Systematic Review

We screened 1419 titles in PubMed and Embase after the first research analysis using the above-mentioned MESH words (Figure 1). After the first search, 1014 abstracts were evaluated and 902 were excluded either because the data were derived from the ELSO register (reason 4) or because they were not pertinent to our objective (reason 5).

Twenty-one full-text articles were assessed for eligibility. Four were further excluded because they did not report specific clinical data on individual patients (reason 6).

Therefore, only 16 articles with a total of 45 patients were included in the review (shown in Figure 1 and Table 1) [19,20,21,22,23,24,25,26,27,28,29,30,31,32,33,34].

### 6.2. Our Experience

During the study period, from 2019 to 2022, there were eight newborns in our center who underwent ECMO for respiratory failure (Table 2).

### 6.3. Mortality Rate

The overall combined survival was 79%, with 87.5% (7 of 8 patients) in our study population and 71% (35 of 45 cases) as reported in the literature. 

### 6.4. Survival Rate in Relation to the Duration of ECMO

The mean duration of ECMO treatment in our study population was 11 days. The main duration of ECMO treatment reported in the literature was 58 days.

Out of the eight newborns included in our study, only one newborn died (patient 1). This newborn underwent a long treatment duration of 41 days compared to the rest of the cohort [35] for whom the mean duration of the ECMO treatment was 6.7 days (patients 2–8).

**Figure 2:**
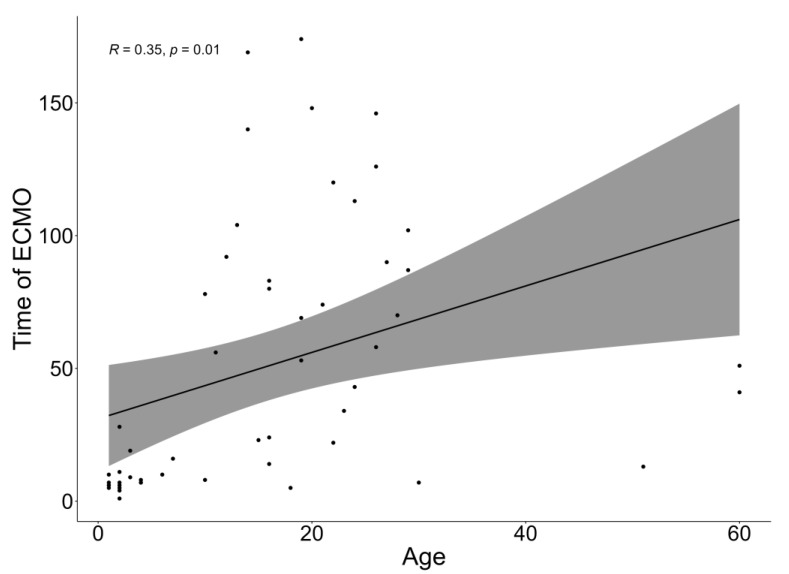
Correlation graph between patient age and ECMO duration of all patients.

As shown in Figure 2, there was a weak positive correlation (R = 0.35) between patients’ age and the duration of ECMO. As the graph demonstrates, the younger the patient is, the shorter the duration of ECMO is. 

**Figure 3:**
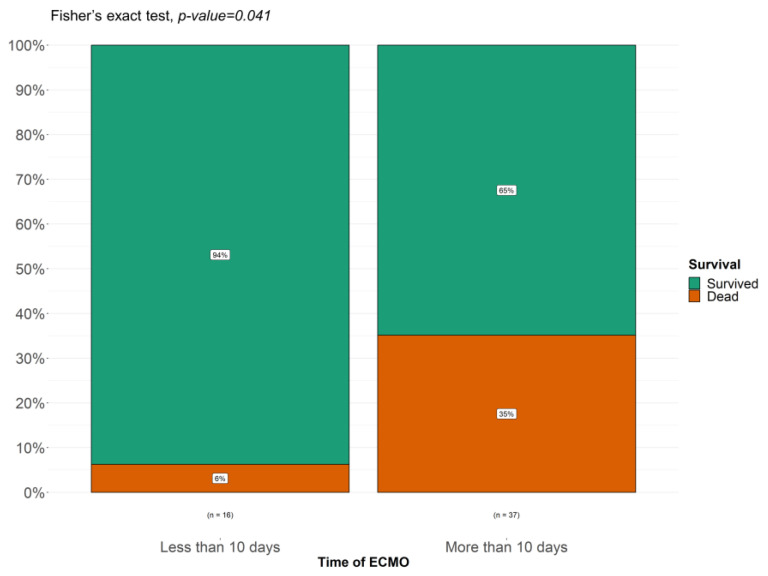
Fisher’s test comparing survival in patients who underwent ECMO for less than 10 days or more than 10 days.

As shown in Figure 3, there is a positive correlation between a shorter duration of ECMO and increased survival. A length of ECMO of less than 10 days is associated with a 94% probability of survival, while a length of ECMO treatment of more than 10 days decreases survival to 65%.

**Figure 4:**
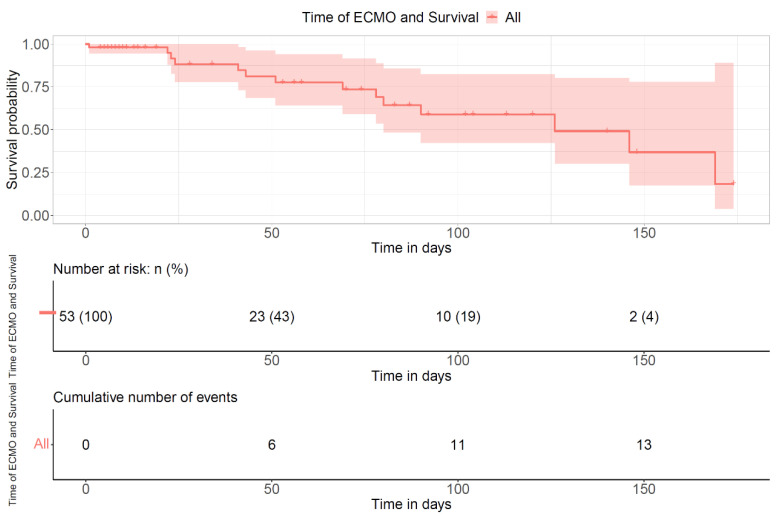
Survival curve of all patients based on ECMO duration.

The survival curve in Figure 4 demonstrates the likelihood of survival based on the length of ECMO treatment. The “events” demonstrate patients who died while on ECMO.

### 6.5. Survival Rate in Relation to the Amnestic Date

The mean patient age at the time of ECMO initiation was 16.8 days in the newborns extrapolated from the literature review and 9.9 days in the study cohort from our center. Our study cohort included one infant older than 60 days (patient 1 [35]). The average age for the remaining patients (numbers 2–8) in our cohort was 2.7 days. There was no statistically significant correlation between age and survival rate.

Five patients from the literature review did not have specified gender [25,32], and of the remaining 40 patients, 62% (25/40) were males [19,22,23,28,30,31,33,34]. Of our eight patients, only one was female [35].

The survival rate in the newborns described in the literature was 76% for male infants and 60% for female infants. With the limitation of small numbers, this demonstrates a trend toward increased survival in male patients compared to female patients. We cannot correlate this with our cohort since we only had one female infant enrolled in the study and she did not survive (patient 1, [35]).

### 6.6. Survival Rate in Relation to the Most Frequent Reasons for Respiratory Failure 

The causes of respiratory failure requiring ECMO in our study and the literature are shown in Table 1 and Table 2.

**Figure 5:**
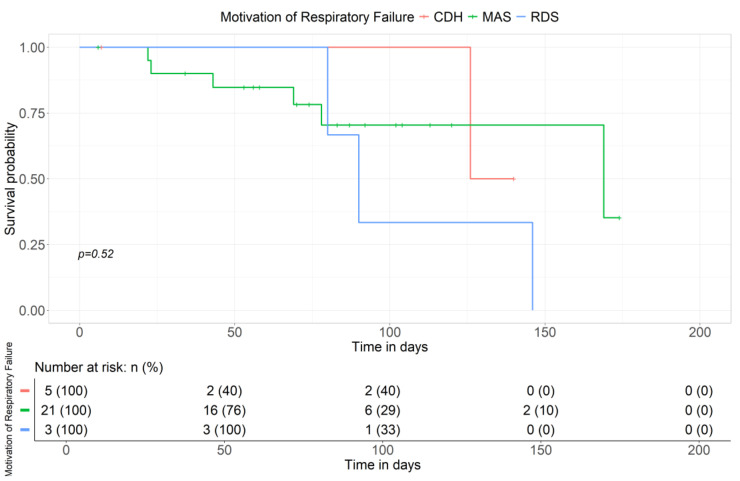
Survival curve in relation to the most frequent reasons for respiratory failure based on literature data.

The graph shown in Figure 5 illustrates the effect of the increased length of ECMO and the etiology of respiratory failure on survival. On day 75 of ECMO treatment, the probability of survival seems to be lower in patients with MAS. On treatment day 150, the probability of survival in infants with congenital diaphragmatic hernia (CDH) and respiratory distress syndrome (RDS) was trending down, while the survival rate for MAS remained unchanged. However, after applying Fisher’s test for MAS and CDH, there was no statistically significant impact on survival based on the underlying etiology (Figure 5).

### 6.7. Survival Rate in Relation to Comorbidities

The comorbidities found in our study cohort were prematurity (two of eight patients) and coinfection (one of eight patients). In particular, patient 1 was born extremely premature, presented with two infections (SARS-CoV-2 and Klebsiella Pneumoniae), and was the only patient in our series who died, while the other premature newborn (patient 5) had no co-infections and survived.

In the literature, comorbidities were identified in 31% of newborns. The remaining 69% of reported infants did not have comorbidities reported. The comorbidities of the patients from the literature review are indicated in Table 1.

**Figure 6:**
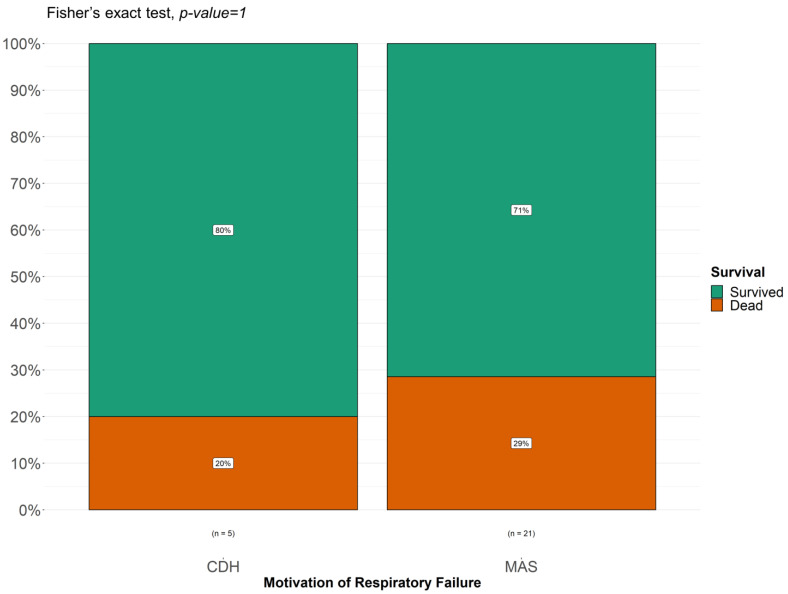
Fisher’s test applied to the most common reasons for respiratory failure.

**Figure 7:**
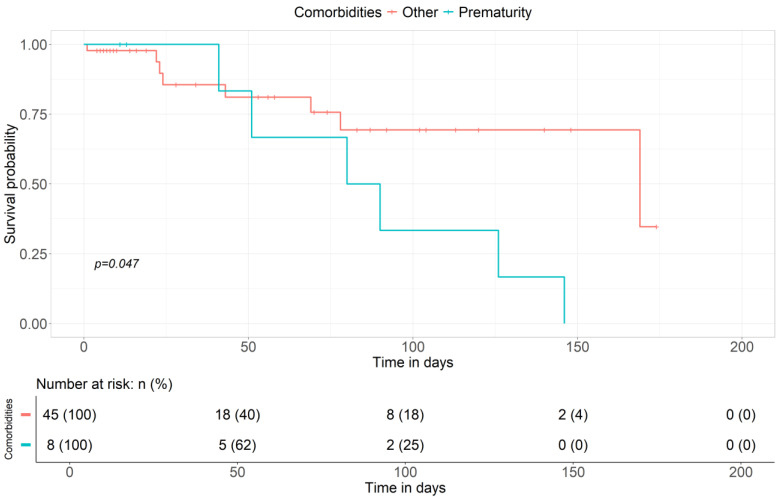
Survival curve in relation to comorbidities, particularly prematurity versus all others.

The most frequently reported comorbidity in the literature (5 cases out of 45) and confirmed in our case series (2 out of 8 patients) was prematurity. The probability of survival in premature infants seems to be lower compared to other comorbidities (shown in Figure 6 and Figure 7). 

**Figure 8:**
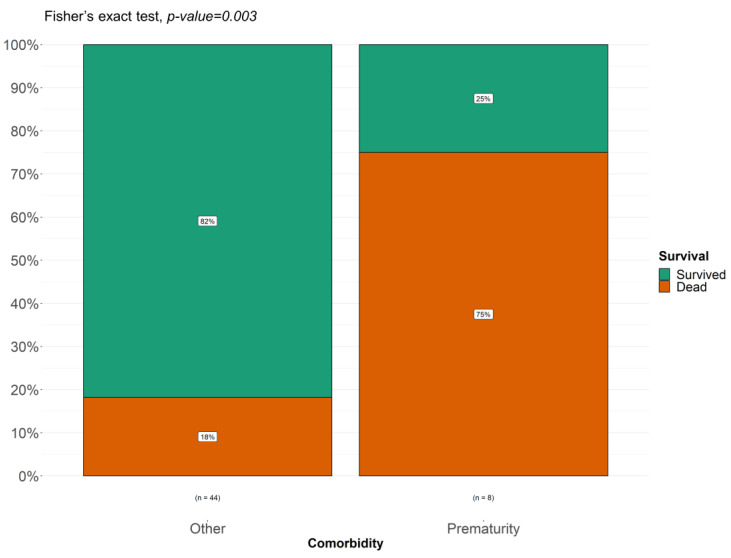
Fisher’s test to compare prematurity with other comorbidity.

Fisher’s test confirmed a statistically significant correlation between prematurity and increased mortality during ECMO (shown in Figure 8).

The other comorbidities are different between works so it is not possible to perform a statistical analysis. However, Sainathan et al. [31] reported the case of a newborn who died during ECMO treatment with the following comorbidities: cardiac surgery and co-infection with SARS-CoV-2 and Candida. This reported case and our patient are the only two cases who died during ECMO treatment and who had co-infection with the SARS-CoV-2 virus. However, given only two cases and the presence of other comorbidities, no conclusions on the impact on survival can be drawn.

### 6.8. Incidence and Correlation of Complications with Mortality

Complications were identified in 44% of cases reported in the literature (Table 2).

The presence of a single complication in 12/20 of newborns reported in the literature was not associated with an overall increase in mortality unless the complication was a hemorrhage. Hemorrhage was the only complication in three patients, of which two died.

The co-presence of two or more complications is a negative predictive factor for survival. In our cohort, the only non-surviving infant suffered from two complications—hemothorax and cerebral hemorrhage (patient 1 [35]). Among the infants reported in the literature, 87.5% of patients (seven out of eight infants) who presented with two or more complications died.

## 7. Discussion

In our rigorous examination, we ascertained the efficacy of ECMO as an instrumental adjunct for respiratory failure, exhibiting an impressive survival rate of 87.5% (seven of eight cases) [35]. However, an evident decrease in survival to 71% was observed for those neonates undergoing revision. All neonates within our cohort exhibited severe lung afflictions precipitating their respiratory failure, further highlighting the indispensable role of ECMO in these scenarios [35].

Navigating through the complexities of ECMO application for neonates, it was discerned that a predominant number faced diverse pulmonary complications. The incidence of pulmonary hemorrhage in six infants, compounded with instances of intense pulmonary hemorrhage and pronounced SARS-CoV-2 infections in others, underscored the multifaceted challenges innate to ECMO treatments in neonates [35]. The premature neonate in our study, whose corrected gestational age was 39 weeks and 4 days at the time of ECMO initiation, unfortunately, succumbed due to severe COVID-19 pneumonia compounded by a Klebsiella pneumoniae infection [35].

Literature reviews provide corroborative evidence on the inherent challenges associated with ECMO in neonates. Cicek et al.’s report described the tragic trajectory of a 16-day-old neonate afflicted with aortic coarctation and SARS-CoV-2 infection, leading to a mortality event after 24 days on ECMO due to sepsis and multiorgan failure [22]. Conversely, Sainathan et al. documented a more optimistic trajectory of a term neonate, 10 days old with trisomy 21, who successfully recuperated after 8 days on ECMO despite suffering from acute respiratory failure resulting from COVID-19 [31]. Both our study and the extant literature signify that prolonged ECMO durations tend to negatively impact survival outcomes. Concomitant complications arising from elongated ECMO durations, encompassing hemorrhages, coagulation irregularities, and specifically, the high risk of intracranial hemorrhage, further accentuate the intricacies associated with this intervention [36,37,38,39,40,41,42].

Despite its myriad insights, our study is not without its limitations.

Our study includes articles from different points in the history of ECMO, ranging from 1994 to the present. Bartlett et al., 1982, is the main contributor with 26 patients (out of a total of 45) [19]. Their results weigh heavily on our data.

Another limitation of our work is the lack of a meta-analysis. It was not possible to perform it because the variables considered in our work are different and, therefore, the sample is significantly reduced.

The salient constraint pertains to the constrained sample size, which impedes our capacity to ascertain ECMO survival factors with the exception of prematurity [35]. Inconsistencies in the extant literature regarding ECMO duration, initiation age, and root respiratory insufficiency further convolute our interpretations. Nonetheless, the pivotal strength of our research lies in its exhaustive literature review. This review discernibly elucidated a strong correlation between mortality rates and the concomitant emergence of two or more complications, with intracranial hemorrhage being particularly detrimental even when manifested solitarily [35].

## 8. Conclusions

We can conclude that although ECMO provides life support in infants with respiratory failure, the rates of mortality and complications remain significant.

Comorbidities are important negative factors that should be considered when predicting the success of ECMO therapy, specifically ECMO duration (more than ten days), gender (female), prematurity, and co-infections. It is necessary to collect more cases to ensure more reliable data.

## Figures and Tables

**Table 1:** Data extrapolated from the systematic review (n = 45 patients).

Authors, Year of Publications	Number of Patients	Age ECMO (Days)	Gender	Comorbidity	Motivation of Respiratory Failure	ECMO Mode	Cannulation Site	Time ECMO (Days)	Survived	Other
Bartlett RH et al., 1982, [19]	1	19	M	ND	MAS	VA	CA, RIJV	69	D	ICB, S, DI
	1	22	F	ND	MAS	VA	CA, RIJV	22	D	ICB, Di
	1	19	M	ND	MAS	VA	CA, RIJV	53	SU	ND
	1	10	M	ND	MAS	VA	CA, RIJV	78	D	S, DI, BL, BE,
	1	14	M	ND	MAS	VA	CA, RIJV	169	D	S, BL
	1	15	F	ND	MAS	VA	CA, RIJV	23	D	ICB
	1	24	F	ND	MAS	VA	CA, RIJV	43	D	ICB
	1	21	F	ND	MAS	VA	CA, RIJV	74	SU	ND
	1	11	F	ND	MAS	VA	CA, RIJV	56	SU	ICB, S
	1	29	M	ND	MAS	VA	CA, RIJV	87	SU	ND
	1	12	M	ND	MAS	VA	CA, RIJV	92	SU	S
	1	26	M	ND	MAS	VA	CA, RIJV	58	SU	S
	1	16	F	ND	MAS	VA	CA, RIJV	83	SU	ND
	1	29	M	Perinatal asphyxia	MAS	VV	RIJV	102	SU	S
	1	24	F	ND	MAS	VV	RIJV	113	SU	BL
	1	13	M	Congenital Myopathy	MAS	VA	CA, RIJV	104	SU	ND
	1	23	M	ND	MAS	VA	CA, RIJV	34	SU	ND
	1	19	M	ND	MAS	VA	CA, RIJV	174	SU	S
	1	28	M	ND	MAS	VA	CA, RIJV	70	SU	BL
	1	22	M	ND	MAS	VA	CA, RIJV	120	SU	ND
	1	20	F	ND	PPHN	VA	CA, RIJV	148	SU	ND
	1	26	F	Prematurity	CDH	VA	CA, RIJV	126	D	S
	1	14	M	ND	CDH	VA	CA, RIJV	140	SU	ND
	1	16	F	Prematurity	RDS	VA	CA, RIJV	80	D	ICB, Diss
	1	27	M	Prematurity	RDS	VA	CA, RIJV	90	D	ICB, S
	1	26	M	Prematurity	RDS	VA	CA, RIJV	146	D	B, DI, ICB
Belousova T, et al., 2019; [20]	1	60	F	Prematurity	RSV	VA	ND	51	D	ND
Cardona V, et al., 2022; [21]	1	3	F	ND	PHE	VA	ND	9	SU	ND
Cicek et al., 2022; [22]	1	16	M	Neonatal congenital heart surgery; candida and SARS-CoV-2 infections	SARS-CoV-2 pneumonia	VA	RA-AA	24	D	ND
Costa J. et al., 2020; [23]	1	2	M	ND	MAS	VA	CA, RIJV	6	SU	ND
	1	7	M	ND	CDH, PH	VV	RA	16	SU	B
Croes F et al., 2010; [24]	1	6	F	Meconium-stained amniotic fluid	CTF	VV	ND	10	SU	ICB
Cuevas Guamán M et al., 2018, [25]	1	1	ND	ND	CDH	VA	ND	6	D	ND
	1	1	ND	ND	CDH	VA	ND	6	D	ND
	1	2	ND	ND	CDH	VA	ND	7	D	ND
Gatzweiler E, et al., 2018, [26]	1	1	F	Lower urinary tract obstruction	PH	VV	ND	10	SU	ND
Hirschl et al., 1994, [27]	1	3	F	Meconium-stained amniotic fluid	Early-onset Listeria monocytogenes infection,	VA	ND	19	L	B
Li G, et al., 2019, [28]	1	2	M	ND	HMD, RDS	VA	ND	28	SU	ND
Raffaeli G, et al., 2017, [29]	1	51	F	Prematurity NEC	Influenza A (H1N1) virus	VA	ND	13	SU	ND
Reiterer F, et al., 1994, [30]	1	16	M	ND	Chickenpox pneumonia	VA	ND	14	SU	ND
Sainathan et al., 2021, [31]	1	10	M	trisomy 21—coarctation of the aorta—SARS-CoV-2 infections	SARS-CoV-2 pneumoniae	VA	RIJV-CA	8	SU	ND
Smith C, et al., 2000, [32]	1	2	ND	Seizures	Pertussis	VA	ND	1	D	ND
	1	1	ND	Pneumothorax hypertension	Pertussis	VA	ND	7	SU	ND
Stammers A, et al., 1997, [33]	1	30	M	ND	RDS	VA	ND	7	SU	ND
Toledo Del Castillo B, et al., 2016, [34]	1	18	M	ND	RSV	VA	ND	5	SU	ND

BE = brain edema; BL = blood leak; CA = carotid artery; CDH = Congenital Diaphragmatic Hernia; CTF = Congenital tracheobiliary fistula; D = death; Di = dialysis; Diss = dissection; ECMO = extracorporeal membrane oxygenation; F = female; HMD = Hyaline Membrane Disease; ICB = intracranial bleeding; M = male; MAS = Meconium Aspiration Syndrome; ND = No Data; NEC = necrotizing enterocolitis; PH = Pulmonary Hypoplasia; PHE Pulmonary Hemorrhage; PPHN = persistent pulmonary hypertension of the newborn; RDS = Respiratory Distress Syndrome; RIJV = right internal jugular vein; RSV = respiratory syncytial virus; S = seizure; SARS-CoV-2: severe acute respiratory syndrome coronavirus 2; Su = survival; VA = venoarterial; VV = venovenous.

**Table 2:** Data extrapolated from a single-center retrospective cohort study (eight patients).

Initials of the Name and Surname	Age in Days	Gender	Comorbidity	Motivation of Respiratory Failure	ECMO Mode	Cannulation Site	ECMO Duration in Days	Survived	Other
1	60	F	Prematurity (27 + 3 GA), Klebsiella Pneumoniae and SARS-CoV-2 infections	SARS-CoV-2 infection	VA	CA, RIJV	41	No	cerebral hemorrhage, Pulmonary Haemorrhage
2	2	M	-	PHE	VA	CA, RIJV	5	Yes	-
3	2	M	-	PHE	VA	CA, RIJV	4	Yes	cerebral hemorrhage
4	4	M	-	PHE	VA	CA, RIJV	7	Yes	-
5	2	M	Prematurity (30 GA)	Perinatal asphyxia	VA	CA, RIJV	11	Yes	-
6	4	M	-	PHE	VA	CA, RIJV	8	yes	-
7	4	F	-	PHE	VA	CA, RIJV	7	Yes	-
8	1	M	.	PPHN	VA	CA, RIJV	5.	Yes	-

VA = venoarterial; VV = venovenous; RA = Right atrium; AA = ascending aorta; ND = No Data; Fem = femoral; ACA = anterior cerebral artery; CA = carotid artery; ECMO = extracorporeal membrane oxygenation; Jug = jugular; RIJV = right internal jugular vein; M = male; F = female, PHE = Pulmonary Hemorrhage; SARS-CoV-2: severe acute respiratory syndrome coronavirus 2; PPHN = persistent pulmonary hypertension of the newborn.

## Data Availability

Data are in the possession of the corresponding author and are available upon request due to privacy or ethical restrictions.

## Supplementary Materials

The following supporting information can be downloaded at: https://www.mdpi.com/article/10.3390/children11121441/s1, File S1: PRISMA 2020 Checklist.
